# Are Surgeons Going to Be Left Holding the Bag? Incisional Hernia Repair and Intra-Peritoneal Non-Absorbable Mesh Implant Complications

**DOI:** 10.3390/jcm13041005

**Published:** 2024-02-09

**Authors:** Andrew W. Kirkpatrick, Federico Coccolini, Matti Tolonen, Samual Minor, Fausto Catena, Andrea Celotti, Emanuel Gois, Gennaro Perrone, Giuseppe Novelli, Gianluca Garulli, Orestis Ioannidis, Michael Sugrue, Belinda De Simone, Dario Tartaglia, Hanna Lampella, Fernando Ferreira, Luca Ansaloni, Neil G. Parry, Elif Colak, Mauro Podda, Luigi Noceroni, Carlo Vallicelli, Joao Rezende-Netos, Chad G. Ball, Jessica McKee, Ernest E. Moore, Jack Mather

**Affiliations:** 1 Regional Trauma Services, Department of Surgery, Critical Care Medicine, University of Calgary, Calgary, AB T2N 2T9, Canada; 2 TeleMentored Ultrasound Supported Medical Interventions (TMUSMI) Research Group, University of Calgary, Calgary, AB T3H 3W8, Canada; 3 General, Emergency and Trauma Surgery Department, Pisa University Hospital, 56124 Pisa, Italy; federico.coccolini@gmail.com; 4 Emergency Surgery Department, HUS Helsinki University Hospital, 00029 Helsinki, Finland; matti.tolonen@hus.fi; 5 Department of Surgery and Critical Care Medicine, Dalhousie University, Halifax, NS B3H 4R2, Canada; sam.minor@nshealth.ca; 6 Head Emergency and General Surgery Department, Bufalini Hospital, 47521 Cesena, Italy; faustocatena@gmail.com (F.C.); carlo.vallicelli@auslromagna.it (C.V.); 7 Surgery Department, ASST Cremona, 26100 Cremona, Italy; andrea.celotti@asst-cremona.it; 8 Department of Surgery, Londrina State University, Londrina 86038-350, Brazil; emanuelgoisjr@me.com; 9 Department of Emergency Surgery, Parma University Hospital, 43125 Parma, Italy; gennaro.perrone82@gmail.com; 10 Chiurgia Generale e d’Urgenza, Osepedale Buffalini Hospital, 47521 Cesna, Italy; giuseppe.novelli@auslromagna.it; 11 Hospital Infermi Rimini, 47923 Rimini, Italy; lucagarulli@gmail.com (G.G.); luigi.noceroni@auslromagna.it (L.N.); 12 4th Department of Surgery, Medical School, Aristotle University of Thessaloniki, General Hospital “George Papanikolaou”, 57010 Thessaloniki, Greece; telonakos@hotmail.com; 13 Letterkenny University Hospital, F92 AE81 Donegal, Ireland; michaelesugrue@gmail.com; 14 Unit of Emergency Minimally Invasive Surgery, Academic Hospital of Villeneuve-Saint-Georges, 91560 Villeneuve-Saint-Georges, France; desimone.belinda@gmail.com; 15 Emergency and General Surgery Unit, New Santa Chiara Hospital, University of Pisa, 56126 Pisa, Italy; dario.tartaglia@unipi.it; 16 Gastrointestinal Surgery Unit, Helsinki University Hospital, Helsinki University, 00100 Helsinki, Finland; hanna.lampela@hus.fi; 17 GI Surgery and Complex Abdominal Wall Unit, Hospital CUF Porto, Faculty of Medicine of the Oporto University, 4200-319 Porto, Portugal; med1873@gmail.com; 18 San Matteo Hospital of Pavia, University of Pavia, 27100 Pavia, Italy; aiace63@gmail.com; 19 Department of Surgery and Medicine, Schulich School of Medicine and Dentistry, Western University, London, ON N6A 3K7, Canada; neil.parry@lhsc.on.ca; 20 Samsun Training and Research Hospital, University of Samsun, 55000 Samsun, Turkey; elifmangancolak@hotmail.com; 21 Department of Surgical Science, University of Cagliari, 09124 Cagliari, Italy; mauropodda@ymail.com; 22 Trauma and Acute Care Surgery, General Surgery, St. Michael’s Hospital, University of Toronto, Toronto, ON M5T 1P8, Canada; joao.rezende-neto@unityhealth.to; 23 Acute Care, and Hepatobiliary Surgery and Regional Trauma Services, University of Calgary, Calgary, AB T2N 1N4, Canada; ball.chad@gmail.com (C.G.B.); jpmather@gmail.com (J.M.); 24 Ernest E Moore Shock Trauma Center at Denver Health, Denver, CO 80204, USA; ernest.moore@dhha.org

**Keywords:** incisional hernia, ventral hernia, mesh, complications, enteroprosthetic fistula, regulatory oversight

## Abstract

Ventral incisional hernias are common indications for elective repair and frequently complicated by recurrence. Surgical meshes, which may be synthetic, bio-synthetic, or biological, decrease recurrence and, resultingly, their use has become standard. While most patients are greatly benefited, mesh represents a permanently implanted foreign body. Mesh may be implanted within the intra-peritoneal, preperitoneal, retrorectus, inlay, or onlay anatomic positions. Meshes may be associated with complications that may be early or late and range from minor to severe. Long-term complications with intra-peritoneal synthetic mesh (IPSM) in apposition to the viscera are particularly at risk for adhesions and potential enteric fistula formation. The overall rate of such complications is difficult to appreciate due to poor long-term follow-up data, although it behooves surgeons to understand these risks as they are the ones who implant these devices. All surgeons need to be aware that meshes are commercial devices that are delivered into their operating room without scientific evidence of efficacy or even safety due to the unique regulatory practices that distinguish medical devices from medications. Thus, surgeons must continue to advocate for more stringent oversight and improved scientific evaluation to serve our patients properly and protect the patient–surgeon relationship as the only rationale long-term strategy to avoid ongoing complications.

## 1. Introduction

Each year, more than 20 million hernia repairs are performed around the world. Moreover, the costs associated with these procedures are expected to reach almost USD 6.5 billion by 2027 [1]. While inguinal hernias occur most frequently, ventral incisional hernias are particularly common and uniquely problematic [2]. Indeed, in high-risk patients, this condition can be expected to occur after the index laparotomy more than 40% of the time [3,4,5,6]. Adding further complexity is that recurrence rates following repairs of these hernias can be almost 20% [7]. Thus, repair of ventral incisional hernias is frequently complicated by recurrence and, clearly, the perfect operation has yet to be found. While many patients opt not to have their hernia repaired, many others undergo different operations with varied success; indeed, many repairs often fail, leading to yet further operative interventions [8]. Ultimately, a small incisional hernia that has been the initial event can cascade into abdominal wall failure, with loss of domain of the viscera, and leaving the patient an “abdominal wall cripple.”

Beginning in the late 20th century, there was increasing evidence that hernia mesh improved outcomes in management of groin hernias. As a result, more and more frequently mesh was also being used to manage ventral hernias, although this strong recommendation had very low evidence [8]. The remainder of this discussion will be specific to the use of mesh for ventral or incisional hernias. Surgical mesh is a medical device that supports the repair of a hernia as it heals. The use of mesh decreases hernia recurrences and has thus become standard practice [9,10,11,12,13,14,15,16,17,18], and has even be considered prophylactically when closing an incision at the first laparotomy [19,20]. The vast majority of patients are greatly benefited by the use of mesh, and it would be hard, if not impossible, to practice hernia surgery currently without mesh except at specialized centers or in low resource settings. Indeed, a number of different techniques have been described for the management of these hernias and for the placement of mesh. Thus, every incisional hernia repair now requires this dual choice, merging a surgical technique to a surgical implant choice.

Like almost anything in medicine, however, mesh hernia repair has a small but constant complication rate, with consequences ranging from inconvenient to devastating. Prompt and diligent attention of the surgeon can often mitigate the affects on the patient. Prompt and skillful post-operative care can rescue many mesh complications. Thus, all surgeons must be familiar with these complications and the strategies to address them. Realistically, contemporary hernia surgery is now practiced under the bright lights of medicolegal challenges and social media misinformation, and this is then combined with a bewildering array of mesh choices, providing for significant confusion amongst practitioners. Yet, despite this context, there is a distinct lack of regulatory oversight. Thus, any thorough discussion of managing complications in ventral incisional hernia must not only consider the operating room and the post-operative wards, but also the courtroom many years hence. We thus hope to comprehensively review the known complications and management options for incisional hernia repair, while highlighting areas in need of further research and understanding, specifically including the regulatory background for existing and potentially new mesh adjuncts.

## 2. Surgical Mesh

Historically, ventral hernia repair has been a challenging operation for the patient with a recurrence rate often exceeding 50% [21,22,23,24]. However, augmentation of the primary tissue repair with a reinforcing mesh may decrease these recurrence rates to between 2 and 36% [4]. Luijenijk’s randomized trial comparing mesh repair to primary suture repair of ventral hernias demonstrated a nearly doubled recurrence rate with suture repair alone at a three-year follow-up [25]. In this study, the prosthetic mesh was sutured to the dorsal side of the fascia with either the peritoneum closed, the omentum sutured between, or an absorbable polyglactin mesh interposed between the prosthetic mesh and the viscera [25]. These authors subsequently followed their mesh-repaired patients for an average of 98 months and noted that, while the recurrence rate in the mesh group was half the suture repair rate, 17% of mesh-repaired patients had a repair related complication which consisted of small bowel obstructions (12%), fistula from mesh to skin (5%), infected mesh (2%), and enterocutaneous fistula (3%) [26]. Thus, even in this well-designed study, conclusions regarding the use of mesh, method of mesh of placement, and whether mesh is even appropriate remain complex. There thus remains a lack of objective data and much subjective opinion regarding the appropriate use of mesh in incisional hernia repair.

### 2.1. Mesh Classifications

In theory, surgical mesh is meant to achieve physical integrity of the components of the musculofascial layers of the abdominal wall equivalent to the native structures [22]. An ideal mesh should be non-toxic, have sufficient mechanical strength and stable physical and chemical properties, ease of handling without displacement, anti-adhesive and anti-infective properties, and it should be cost effective [17,22]. To date, the ideal mesh does not exist. 

As the science continues to advance, there are now many different manufacturing processes for mesh. In addition, numerous attempts have been made to classify mesh types; from simple to complex [22]. At perhaps the most basic level, mesh can be classified into absorbable and non-absorbable. In evaluation of prosthetic meshes, they can also be classified by mesh weight, pore shape, and pore size [16,22]. Prosthetic meshes may also be differentiated as to whether they are reticular, laminar, or composite and whether they are knit or woven [1,16]. A common system is to classify synthetic mesh by porosity. Type I is considered to be “macroporous” with pore size > 10 microns; type II is “microporous” with pore size < 10 microns; and type III is a composite of both micro- and macroporous elements. Nearly all synthetic nondegradable meshes are made of polypropylene, polyvinylidene fluoride (PVDF), polyethylene terephthalate polyester, or expanded polytetrafluoroethylene (ePTFE) [16]. Composite meshes are made of two or more components and typically require a specific orientation with placement. They contain a traditional mesh component which will permit tissue ingrowth as well as a protected peritoneal side with a non-adherent mesh surface or surface coating [2,16,22,27,28,29]. Reticular meshes allow better ingrowth of cells between their fibers, while lamellar prostheses such as PTFE do not support cellular ingrowth within their substance [1]. Thus, PTFE meshes have been associated with poor resistance to infection as white blood cells are prevented from accessing mesh [30]. Alternatively, polypropylene has been developed with larger pore sizes and lower density. These two factors allow easier ingrowth of native tissue and vascularization which increases the resistance to infection [1]. Some macroporous prostheses contain pore sizes greater than 75 microns, which is large enough to allow ingress of cellular fibroplasia and angiogenesis. A totally microporous mesh has pores less than 10 microns in at least one dimension and can thus resist cellular ingrowth [31]. Polypropylene is the most common hernia mesh used globally but is known to cause dense adhesions to any bowel to which it is exposed [16,17,22,23]. Further, the heavy-weight mesh may be associated with chronic pain from a profound foreign body response and fibrosis in both ventral and inguinal hernias [16,32]. 

### 2.2. Biological Meshes

Biological prostheses (biological meshes or bioprosthetic materials) are classically used for complex or contaminated abdominal hernia repairs, as they may cause less inflammation and fibrosis than synthetic meshes, making them suitable for infected or potentially infected fields [33,34]. They are typically derived from human (allogenic) or animal tissues (xenogenic), such as porcine or bovine, and processed to remove cellular components, leaving behind a collagen scaffold [35]. In theory, biological meshes are designed to integrate with the patient’s own tissue over time, potentially leading to a more natural and durable repair, which may result in fewer complications, especially in a contaminated field [34]. The use of biological mesh, however, comes at a high economic cost; these meshes can cost up to 200 times more than synthetic mesh. Indeed, there remain many questions regarding surgical technique, long-term outcomes, and health economics with respect to the use of biological mesh. A well-performed multi-centre randomized trial comparing synthetic versus biological mesh in contaminated ventral hernia fields (with a retromuscular placement) reported a recurrence rate nearly 4 times higher in the biological mesh group but no difference in risk of surgical site infectious complications between groups at 2-year follow-up. Moreover, the median cost of the biologic mesh was $21,539 vs. $105 for the synthetic mesh. [36]. However, intraperitoneal placement of biological meshes has not been associated with the same long-term complications as non-biological prosthetic intraperitoneal mesh placement, as we shall see below.

### 2.3. Anatomic Review of Mesh Placement

**Figure 1:**
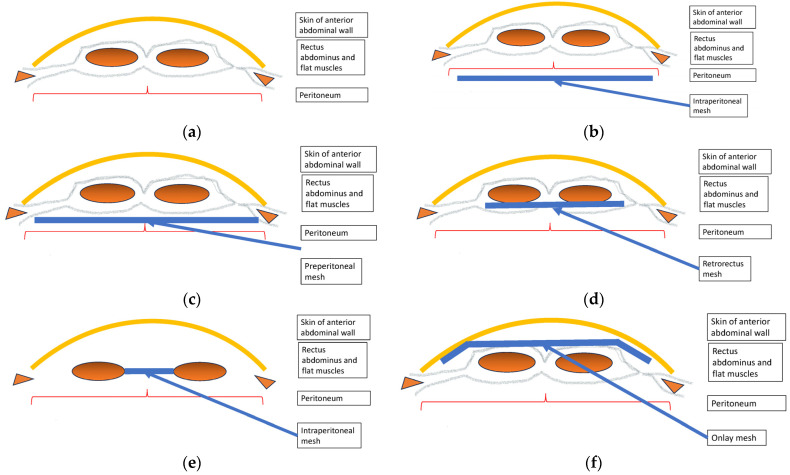
Schematic diagram of anatomic abdominal incisional hernia mesh placement locations. (**a**) Normal Abdominal Wall. (**b**) Intraperitoneal mesh. (**c**) Preperitoneal mesh. (**d**) Retrorectus mesh. (**e**) Inlay mesh. (**f**) Onlay mesh.

Surgical meshes may be implanted into a number of anatomic positions in the anterior abdominal wall (Table 1) (Figure 1). These positions constitute the intra-peritoneal, preperitoneal, retrorectus, inlay, and onlay positions [10,24,37]. These will be discussed below.

#### 2.3.1. Intra-Peritoneal Placement of Mesh

These meshes are intended to be implanted within the peritoneal cavity proper and are, therefore, in direct contact with the intra-abdominal viscera. These devices may utilize anti-adherent physical barriers and can include prosthetic-coated, composite-coated, or biological [1]. There are also examples of intra-peritoneal non-coated synthetic meshes that are protected from visceral adhesions by interposing omentum with reportedly acceptable results in uncontrolled series [38]. 

#### 2.3.2. Intra-Peritoneal Onlay Mesh (IPOM) Placement

Since the introduction of minimally invasive ventral hernia repair which consists of an intra-peritoneal onlay mesh (IPOM) technique, there has been uncertainty as to whether it benefits patients. The technique seems to confidently decrease local wound complications and may shorten hospital stay [23,24,39,40,41]. However, despite a moderate evidence base and numerous randomized controlled trials, there has been no conclusive determination of whether open or laparoscopic techniques, with mesh in an intra-peritoneal position, provides a benefit to patients [39,42,43]. Indeed, a pertinent comment made by the Cochrane review group is that there is a “rare but theoretically higher risk that intraabdominal organs are more likely to be injured during a laparoscopic procedure [39]”. The Italian Laparoscopic Ventral Hernia Guideline group meta-analysis showed that the laparoscopic technique was associated with increased accidental full-thickness enterotomies [9]. Further, a nation-wide population-based review from France concluded that laparoscopic IPOM placement significantly increased the risk of bowel obstruction compared to patients with a previous laparotomy but no intra-peritoneal mesh [18]. The most recent Midline Incisional hernia guidelines from the European Hernia Society also state that any mesh in the abdominal cavity exposed to the abdominal viscera should be used with caution due to the risk of long-term complications at any subsequent abdominal surgery,” and to “keep the mesh out of the peritoneal cavity where possible to limit contact with the viscera” [8].

### 2.4. Complications of Mesh Placement

Complications of intraperitoneal mesh have been generally classified as minor versus major [3]. Minor complications include seromas, hematomas, recurrent pain, and superficial surgical site infections. Major complications include hernia recurrence, complications of subsequent surgery, adhesive bowel obstruction, mesh contraction, deep prosthetic infection (i.e., mesh infection), enterocutaneous fistulae [17,18,22], and protracted medicolegal proceedings (Table 2).

#### 2.4.1. Management of Minor Complications of Incisional Hernia Repair with Mesh

Seromas frequently complicate hernia repairs when the surgical site must be dissected in order to create an anatomic space for mesh implantation. Surgeons have long been taught to liberally use wound drains to prevent post-operative fluid collections and their sequalae, such as wound dehiscence and infection. This practice, however, has not been particularly well studied in the hernia population. [44,45,46,47,48]. While one recent randomized study demonstrated no difference between the size of residual fluid collection between a drain vs. no-drain group, they also demonstrated a significantly lower complication rate in the drainage group, including less risk of dehiscence [46]. When a seroma does occur post-operatively, it can most frequently be managed conservatively and most resolve with time, especially if there are no features suggestive of superimposed infection. If the seroma is symptomatic and persistent, we offer repeat percutaneous aspiration or drainage. As part of the informed discussion with the patient, it is vital to reiterate that there is a small risk of introducing infection with every aspiration. If there are concerns for potential or actual infection, this can typically be confirmed with aspiration of the seroma, most easily done under ultrasound guidance. If a surgical site infection (SSI) is strongly suspected or confirmed, appropriate antibiotics should be administered early for an appropriate length of time according to the clinical response of the wound and ideally directed by culture results. The local microbiological characteristics of the hospital should be known, and infectious disease consultation may be appropriate both to treat the patient properly, but also to prevent overuse of antibiotics and development of antibiotic resistance [49]. If there is purulence or frank pus within a wound, it should be opened, and the wound packed with regular dressing changes. SSIs may or may not involve any contiguous mesh. Exposure or infection within the anatomic compartment containing the mesh intuitively increases the complexity of the problem, and a mesh infection whether acute or chronic constitutes a major complication.

##### Autoimmune Complications Have Not Been Validated

Fortunately, autoimmune reactions to mesh, while dramatized in the lay press after being suggested by a methodologically poor case series [50], have not been scientifically validated [51,52]. Neither has any valid evidence to support male infertility been published [53].

#### 2.4.2. Major Complications of Incisional Hernia Repair with Mesh

##### Complications of Subsequent Surgery

When formulating a plan for repair of a ventral hernia, an important waypoint is to consider any ramifications of the repair on any future operative interventions. For example, mesh placement is associated with more peri-operative complications at a subsequent operation when that mesh is placed in an intra-peritoneal position [4]. Indeed, a review of such outcomes found that, after re-laparotomy, 76% of patients with previous intraperitoneal mesh placement had perioperative complications compared with only 29% in patients with pre-peritoneal mesh. Moreover, in the intraperitoneal mesh group, 21% of patients required a small bowel resection compared with none in the preperitoneal group [4].

##### Mesh Infection

Prosthetic mesh infection (PMI) is often a devastating complication for which there are sparingly few well-controlled scientific studies beyond biased opinion and previous experience [54]. The risk of mesh infection has been reported to be from 1% to as high as 25.6% depending on the technique, patient population, and type of mesh [16,23,28,41,55]. In particular, the incidence of infection depends heavily on mesh selection and surgical technique. Polypropylene meshes have been reported to have infection rates ranging from 2.0 to 4.2%, while ePTFE infection rates may vary from 0.0% to 9.2% [56]. Multifilament polyester meshes show the highest infection rates that may range from 7.0% to 16% [56,57]. Some authors do not consider incisional hernia repair as clean surgical cases owing to marked infection rates in some series [58], although this has not been universally accepted. 

Superficial incisional infections can typically be managed without the need for mesh removal, nor are they influenced by the use or choice of mesh [55]. Oral antibiotic therapy is frequently sufficient for management. However, deep prosthetic infections can have profound deleterious effects. While the initial infection is typically acute, it can be followed by a chronic inflammatory response that may generate further fibrosis, bowel entrapment, and ultimately fistulization with internal or external enterocutaneous fistulae formation. The use of open wound management with negative pressure wound therapy may often be able to salvage an onlay polypropylene prosthetic mesh (see below). Unfortunately, PTFE or dual-coated meshes have been reported to require complete excision and are not amenable to such attempts at conservation due to their innate characteristics [55]. 

#### 2.4.3. Salvage of Infected Mesh

It has been conventionally taught that management of a PMI will mandate removal of the mesh [14,55]. In practice, however, this often equates to multiple reoperations, complex wound care, and the development of a recurrent hernia potentially larger than even the inciting defect [54,59]. Depending on the location and mesh type, it may be possible to salvage some meshes using antibiotics, interventional radiology, conservative surgical debridement, and negative pressure wound therapy [15,55,60]. Warren and colleagues concluded that mesh properties and position within the abdominal wall were the primary determinants regarding salvage of infected mesh. Notably, as demonstrated in one of the largest series of PMI, mesh in an intra-peritoneal position was more frequently associated with infection (58.7% of all PMI) and was rarely salvageable (2.4% of cases) [54]. Moreover, these infections were frequently associated with development of enteroprosthetic fistulae which occurred in 17.8% of cases (53). Macroporous polypropylene mesh was salvaged in 65% of cases (>72% when used extraperitoneally). Microporous mesh, however, was salvaged in only 7.7% of cases (53). When a PMI was associated with a composite or PTFE mesh, none were salvageable. Percutaneous drainage and antibiotics were able to salvage 34.5% of cases, all of which were microporous polypropylene or biological mesh. Local wound care salvaged only 18.8% of meshes, of which 80% were macroporous polypropylene [54]. The potential salvageability of polypropylene related to other prosthetic mesh formulations has been confirmed by others [55,60]. The relative difference in salvageability of a mesh is again related to the sizes of the pores, the weave of the mesh, and its anatomic position. If the pores are large enough to allow white blood cells (WBCs) to enter within the mesh, then bacteria can be eradicated by the body, and conversely if the pores are too small, bacteria may contaminate a mesh and be physically protected as the pore size will not admit leucocytes. A large review of vacuum-assisted closure therapy with infected mesh confirmed the highest salvage with polypropylene mesh (93.5%), intermediate with composite (83.3%), and none with PTFE. Furthermore, onlay (83%) and retromuscular (98.5%) had higher rates of salvage than IPOM (56%) [60].

Although not the most favorable anatomic position biomechanically [24], the onlay placement of a polypropylene mesh facilitates VAC therapy if necessary. Intra-peritoneal mesh placement does not allow for this salvage therapy and may make earlier detection of a mesh infection more difficult leading to a delay in therapy. We, therefore, question the wisdom of placing any prosthetic mesh inside the peritoneal cavity since, when infected, they frequently cannot be rescued and, perhaps even more significantly, can lead to highly morbid intraabdominal sequelae. Warren and colleagues similarly concluded their report on infected prosthetic mesh with the statement “the high proportion of patients in this study with an IPOM technique who developed secondary mesh infection after a subsequent abdominal operation should prompt special consideration of mesh selection and its position within the abdominal wall [54]”. 

When the decision has been made to remove infected mesh, the next question to consider is how much mesh to remove? Bueno-Lledo et al., published a relatively large series comparing complete mesh removal to partial removal for infected prosthetic mesh [55]. Partial mesh removal involved explantation of non-incorporated mesh and was less morbid for the patient. Not unsurprisingly, complete mesh removal led to more hernia recurrence (47.9%) and more frequent and severe post-operative complications, while persistent or recurrent infection was noted more frequently with partial removal [55]. Thus, translating the best evidence still requires surgical experience to balance morbidity versus benefit for every case of mesh infection requiring operation. However, infection is not the most concerning or serious risk of an intra-peritoneal prosthetic mesh.

#### 2.4.4. Mesh Shrinkage, “Meshomas”, and Bowel Obstruction after Incisional Hernia Repair with Mesh

Mesh shrinkage may have radically different implications depending on where a mesh is implanted and whether the mesh relies upon a protective coating to avoid visceral adhesion/erosion. A “meshoma” has recently been defined as the folding or balling up of mesh which contributes to chronic pain, hernia recurrence, and or nerve entrapment [14,61]. After the implantation of any foreign object, the immune system will react with an intensity and chronicity related to the chemical and morphological construction of the mesh [1,2,22,62,63]. It is reported that this can result in seroma formation and encapsulation as well as mesh shrinkage, sometimes by up to 60% or more [22,31,62]. Meshomas related to intra-peritoneal mesh are also associated with bowel entrapment, obstructions, enteroprosthetic and enterocutaneous fistulae [54]. One animal model documented that even with “protected” composite intra-peritoneal polypropylene-based mesh, 40% of animals still developed adhesions despite the protective barriers [2]. While the risk of bowel obstruction from adhesive disease following intraperitoneal violation is well documented, there has been sparse literature evaluating the specific risk of bowel obstruction following ventral hernia repair. It is highly likely that intraperitoneal placement of mesh will increase the risk of adhesion formation and subsequent bowel obstruction and should be taken into serious consideration when determining mesh position. 

#### 2.4.5. Enteroprosthetic and Enterocutaneous Fistula after Incisional Hernia Repair with Mesh

Perhaps the most feared complication of intra-peritoneal mesh placement is that of fistula development; yet, this highly morbid sequela is poorly documented in the medical literature. Fistula creation is facilitated by erosion of the mesh into the surrounding viscera [27]. The large series previously discussed from Warren noted that most (81%) of enteroprosthetic fistulae were associated with IPOM mesh [54]. Patients with enteroprosthetic fistulae tend to present much later; on average 4 years after incisional hernia repair or subsequent surgery. The fact that many of these complications frequently occur many years after implantation renders a five-year period of post-market surveillance for serious complications inadequate to truly understand the health implications of intra-peritoneal mesh. Thus, with a delayed mesh infection occurring years after the index surgery, an enteroprosthetic fistula should strongly be suspected or anticipated [54]. This may be partial thickness such that the mesh is adherent but with complete perforation or may be full-thickness resulting in intestinal perforation. As the result of surrounding inflammation and scarring, this typically does not result in acute intra-abdominal sepsis but rather in a chronic ongoing fistula to the skin and a resultant enterocutaneous fistula. All usual resuscitative and supportive measures for intra-abdominal sepsis may be required to support an acutely sick patient [64]. Standard measures to manage the enterocutaneous fistula are also appropriate in this setting, allowing time to prepare for a definitive solution. The only way to cure this complication is to perform a complete resection of the mesh and involved viscera which may be a very morbid and complex operation. Often elderly or comorbid patients will not be able to tolerate such surgery and a life-long acceptance of this debilitating condition may be the only, albeit suboptimal, solution. Thus, a corollary to the recommendation for IPOM in patients “not fit enough for open surgery” may be the recognition that they will certainly not be fit enough for any reconstructive surgery if required in the future.

#### 2.4.6. Comparative Evidence Supporting the Use of Intra-Peritoneal Mesh for Incisional Hernia Repair

Soare and colleagues recently performed a contemporary systematic review of complications related to the intra-peritoneal placement of mesh [3]. They concluded that this technique lacks rigorous follow-up, thus missing major and previously unforeseen long-term complications. Indeed, more rigorous randomized studies are needed to justify whether to continue with the practice [3]. Notably, joint guidelines from the European and American Hernia Societies do not advise implanting a synthetic mesh prophylactically in the intra-peritoneal space given the increased risk of adhesive complications [19]. After a review of complications occurring with intraperitoneal prosthetic mesh placement, Halm and colleagues concluded that “intra-peritoneal placement of polypropylene mesh at incisional hernia repair should be avoided if possible” and noted that intra-peritoneal meshes were associated with complications in 77% of cases requiring a subsequent relaparotomy [4]. Alternatively, the most recent Italian national guidelines on laparoscopic treatment of ventral hernias recommended laparoscopic surgery with an intra-peritoneal mesh in defects less than 10 cm, in the elderly, obese, and in emergency settings, but noted generally very low evidence and commented that the uncertain risks of an intra-peritoneal prosthesis made all their guidelines conditional [9]. 

## 3. Discussion of the Gaps

### 3.1. The Surgeon–Patient Relationship and Implantable Devices

No matter how complex the manufacturing–evaluation–regulatory infrastructure, it is the individual surgeon and patient who take the irreversible leap of faith to permanently implant a mesh within a human body. Although it is assumed by both that this mesh protects the patient against the distress of a hernia recurrence, it also presents some degree of life-long risk of potential infection, mesh erosion, or mesh migration [4,6,51,65]. It has been noted that the involvement of patients in the decision-making process of embarking on hernia surgery can be limited [66]. Any surgeon involved in hernia surgery and utilizing mesh products in their repair must increasingly be aware of growing medicolegal concerns as well as the growth of patient support groups focused on problems related to the use of “mesh” in their repairs [66]. There is also an ever-increasing level of mistrust between patients and the “surgical industry” in general [66]. Further, the term “mesh-injured” has appeared in the lexicon, although it may functionally encompass many interrelated issues in the conduct of hernia repair that may have no relation to mesh whatsoever [66]. Thus, repairing hernias, long considered “bread and butter” general surgery, has become an increasingly politicized field of surgery where surgeons who simply want the best outcomes for their patients may unwittingly become the “bad guy/gal”. Such challenges to this practice ought to call for increasing data collection and the output of high-quality research to make advising patients simple and logical. Unfortunately, the converse has proven true. Hernia research has unfortunately been referred to as an “oxymoron”. When reviewing all the published studies concerning ventral hernias, less than 3% of published studies were randomized-controlled trials [10,11]. However, any attempt at an organized analysis of surgical techniques is admirably better than the analysis of surgical devices for which there is essentially no research (discussed below).

However, despite some notable efforts, the authors soberly contend that it is a blemish on both the profession and regulators that so little good research has been performed to inform surgeons how to best to treat these patients. Indeed, the most recent combined guidelines from the European and American Hernia societies noted “the limited quantity and/or quality of the studies available to answer key questions” [19]. The frequency of this condition, however, has provided a massive profit-making opportunity for medical device companies who have marketed an array of technical options that have little good science backing them. It is a further shame that regulatory bodies charged with the responsibility to protect patients have largely abandoned this responsibility and require little or no data regarding efficacy to approve medical devices. The authors are increasingly being required to perform complex and morbid abdominal wall repairs involving hernia recurrence, bowel obstructions, and the most feared complication, mesh-incorporated enterocutaneous and enteroprosthetic fistulae. This admittedly anecdotal experience thus prompts us to attempt to understand the Regulatory and Commercial background that complicates the best practice of ventral incisional hernia surgery.

### 3.2. Not Better, Not Even Safe, Just “Substantially Equivalent” (To What?)

The world is rife with unsubstantiated conspiracy theories. Many, for example, still believe that that the earth is flat. While it is certainly true that, historically, pharmaceutical manufacturers have grossly violated human rights and valued corporate profits over human well-being [67], there is now strict oversight of pharmaceutical development and marketing. Contemporary pharmaceutical companies should be complimented for having developed and appropriately tested many life-saving and life-improving drugs [68]. The public can also be reassured that any new pharmaceutical marketed will have undergone a rigorous process of testing and controlled study before being allowed on the marketplace. New pharmaceuticals must undergo Phase I, Phase II, and finally rigorous Phase III prospectively randomized adequately powered trials to conclusively demonstrate their benefit to patients [69].

However, one global conspiracy that actually appears to be a valid concern, and particularly affects the practice of surgery, relates to the release of medical devices for human use. Most surgeons and patients naturally assume that the device to be implanted will have been proven safe and efficacious. Unfortunately, this is not true [70]. Simply, the Emperor has no clothes; and surgeons may be left holding the bag as ignorance is not a valid legal defence. Under most existing regulations, implantable medical devices do not have to be shown to be efficacious, or even safe, but just to be “substantially equivalent” to some other device that has been historically used in surgery [71,72,73,74,75]. This means that as long as some surgeon previously believed that using some device was “a good idea,” any corporation can introduce a new device to the market that is “substantially equivalent” to the older device that was “grandfathered” into practice. The United States Food and Drug Administration (FDA) standards of evidence relate to predicate devices (devices which can be legally marketed and serve as a point of comparison for new devices) marketed as part of interstate commerce prior to May 28, 1976. Beyond this “equivalence”, minimal to no evidence of effectiveness, efficacy, or even usefulness is required to market a medical device in Canada or the United States [74,76]. In the United States, this process is known as the 510 (k) exemption. A “510 (k)” is a “premarket submission made to the FDA to demonstrate that the device to be marketed is as safe and effective, that is, substantially equivalent, to a legally marketed device [77].” With an exemption to this 510 (k) policy, the approval process no longer requires post-market surveillance for complications which ironically seems to put the onus on surgeons to report problems rather than ensuring safety prior to market release. Shah and colleagues recently reported that while few 510 (k)-exempt devices had any published research even 5 years after release, and 10% of these devices were actually subject to recalls [76]. All surgical meshes ever cleared for clinical use have been 510 (k)-exempt and have, therefore, not required any real research [71]. Zargar and Carr reported a remarkable analysis of the regulatory ancestral history of surgical meshes and noted that 97% of meshes introduced between 2013 and 2015, were descended through “substantial equivalence” from only 6 meshes present prior to 1976. Further alarming was the fact that 16% of recently approved meshes were connected through equivalence claims to 3 predicate devices that were actually recalled for flaws causing serious adverse events [71]. This is very concerning as a practicing surgeon will be subjected to a constant barrage of marketing pressure to use new devices with the reassurance that they are “approved”. The result has been the relative uncontrolled proliferation of expensive medical devices marketed as “innovations” with the implied message that if surgeons do not use these devices, they are “laggards”. All practicing surgeons will surely note the great irony that individual manufacturers will emphasize the uniqueness of their own proprietary mesh when advocating for market share, yet twist themselves 180 degrees to emphasize the monotony of similarity with previous mesh when applying for regularity approval. Upon review, Kahan concluded that there was “extreme under-reporting and lack of consistency of clinically important mesh properties” [78]. 

For example, the Kugel Patch consists of a product-line of hernia mesh products introduced in the 1990s. The manufacturer received reports that these devices were failing as early as 2002, but waited almost three years before recalling the mesh [79]. The Composix hernia patch was recalled once it was identified that the recoil ring may break, which could potentially lead to bowel perforation and or chronic enteric fistula [79]. This device was also approved by the FDA 510 (k) “workaround” strategy in 2001 as being “substantially equivalent” to a previous mesh [79]. Ultimately, the manufacturer recalled more than 137,000 of these devices between 2005 and 2007, and paid more than $180 million to settle litigation in 2011 in the United States and $1.4 million to settle related lawsuits in Canada in 2014 [80]. Some of the authors have personally removed entero-prosthetic fistula from our own patients related to this device. A further comment on the confusing regulatory science of recalled meshes on one continent is that they seem to still be available years afterwards on other continents with differing regulations [81,82]. Even more disturbing is that, as Zargar and colleagues have noted, recalled meshes associated with adverse effects may, indirectly, continue to serve as predicates for new devices, thus raising significant concerns over the safety of the regulatory approval process itself [71].

### 3.3. A Global Medicolegal Risk to a Hernia Surgeons

Any surgeon would be naïve to ignore the society within which they practice their craft. Although we have taken oaths to care for our patients and to do no harm, it is impossible to conduct ourselves according to that oath without scientific data. Scientific reports in the medical literature regarding mesh concerns are scant, yet there is an abundant, almost overwhelming amount of medicolegal and opinion advocacy online. Any search of the internet will reveal that the most prominently accessible websites will be those offering to commence legal action by a specially focused hernia mesh lawyer. Although accurate data are not available, the internet would suggest there are more lawyers specializing in litigating hernia mesh lawsuits than there are surgeons specializing in mesh hernia repair. Furthermore, these hernia mesh lawyers seem to enjoy a greater degree of confidence regarding evidence appraisal as they dramatically “inform” patients as to, for example, the symptoms that allergies to mesh produce while soliciting business [83]. Such conclusive but completely science-deprived legal communications contrast starkly with carefully appraised scientific studies that cautiously conclude that “there is little to no evidence that the use of polypropylene mesh can lead to autoimmunity” [51,52]. However, any patient accessing the internet will find the legal advertising rather than the appraised science.

As surgeons, we are taught to obtain our information from peer-reviewed medical journals, and to disregard the mass of “grey literature” or frank dis-information available on the Internet. However, such a purist approach will leave surgeons grossly unaware of the beliefs, understandings, and opinions of the populations we attempt to serve. In Canada, respected news media report that at least 12 brands of hernia mesh have been recalled or removed from the Canadian marketplace since 2000, but PubMed will not reveal this to surgeons. In fairness, the media also accurately reported that the majority of hernia mesh patients have no problems and that data show hernia mesh improves recovery and lowers recurrences [84]. It is thus very easy for patients to access legal websites providing them with some basic facts regarding proprietary meshes that have been removed over mesh-specific concerns. However, finding actual scientific data to better educate surgeons to be experts is impossible as the topic of mesh recall seems to have been ignored by Academia. The British Broadcasting Agency (BBC) has well stated the situation reporting that “currently, hernia mesh devices can be approved if they are similar to older products, which themselves may not have been required to undergo any rigorous testing or clinical trials in order to assess their safety or efficacy” [85]. Further, the BBC further voiced the opinion of the authors that “there is a lot of secrecy surrounding the approval of hernia mesh, with even doctors unable to access the clinical data” [85].

There may be the awakenings of initial consciousness in regulatory agencies, however. In 2014, after product recalls and ongoing compensation litigation, the FDA reclassified synthetic and non-synthetic meshes for pelvic organ prolapse from Class II to Class III devices, meaning that actual research would be required for future meshes in this category. Unsurprisingly, no new such meshes have been introduced since [71]. The authors (who practice hernia repair), believe that in order to enhance the protection of all patients, new devices must be proven safe and that prospective clinical trials must the minimal standard. We further suggest that, given the massive costs of healthcare, the safety of new devices must also be prospectively studied in the context of patient-centric outcomes and, ideally, economics to prove that any new device is actually “better.” Otherwise, why are they needed in the first place?

## 4. Conclusions and Future Directions

Given the immense complexity of the use of mesh for incisional hernia repair, the authors are not able to answer many of the key questions surrounding this topic. We do conclude that prosthetic mesh repairs have benefited many patients globally. We continue to perform prosthetic mesh-augmented incisional hernia repair, but we believe it is prudent to avoid intra-peritoneal placement of any prosthetic mesh until adequate and conclusive scientific studies have been completed. We further warn all surgeons that in the current highly litigious climate, future medicolegal concerns regarding any use of mesh should be anticipated and that current regulatory bodies of many if not most First World nations do not appear to have prioritized the interests of patients, surgeons, or science as part of their framework. It is thus a complex but urgent responsibility for surgeons to try to understand the issues better and to advocate for good scientific data that will vindicate us when the judge states the obvious fact that “doctor, the operative reports clearly records that YOU made the decision to implant this device”.

## Figures and Tables

**Table 1:** Anatomic locations within the anterior abdominal wall utilized for permanent mesh implantation.

Location	Posterior Structures	Anterior Structures	Location-Pros	Location-Cons
Intraperitoneal	Peritoneal cavity	Peritoneum	Biomechanically strong	Adjacent to viscera Inaccessible if infected
Preperitoneal	Peritoneum	Transversalis fascia	Biomechanically strong	Potentially adjacent to viscera (peritoneal defect)
Retrorectus	Posterior Rectus Sheath	Rectus Abdominus Muscle	Biomechanically strong	Limited width of mesh (except TAR ^1^ uses very large mesh)
Inlay	Mesh inlaid between edges of hernia defect with no overlap	Subcutaneous tissue	None	Adjacent to Viscera Biochanically very weak
Onlay	Anterior rectus sheath and External oblique	Subcutaneous tissue	Accessible to local salvage therapies in case of infection Distant from viscera	Less biomechanically strong

^1^ TAR = Transversus abdominus release.

**Table 2:** Mesh complications.

**Minor**
Seroma
Hematoma
Recurrent pain
Surgical site infection
**Not-validated**
Autoimmune reactions
Male infertility
**Major**
Hernia recurrence
Complication of subsequent surgery
Adhesive bowel obstruction
Mesh contraction
Mesh infection
Enteroprosthethic fistula
Enterocutaneous fistula

## Data Availability

No new data were created or analyzed in this study. Data sharing is not applicable to this article.
